# Recurrent Basal Cell Carcinoma (BCC) of the Forearm: A Case Report

**DOI:** 10.7759/cureus.40247

**Published:** 2023-06-11

**Authors:** Safaa Abatli, Mohammed Hasan, Suha B Sholi, Ahmad Qashoo, Iyad Maqboul

**Affiliations:** 1 General Surgery, An-Najah National University Hospital, Nablus, PSE; 2 Plastic and Reconstructive Surgery, An-Najah National University Hospital, Nablus, PSE; 3 Medicine, An-Najah National University, Nablus, PSE; 4 General and Laparoscopic Surgery, An-Najah National University Hospital, Nablus, PSE

**Keywords:** reconstruction after bcc excision, forearm reconstruction, agressive bcc, bcc of forearm, recurrent bcc, perineural invasion of bcc

## Abstract

We describe a case of basal cell carcinoma (BCC) in a 36-year-old lady. She had a history of recurrent BCC in the ventral aspect of her right forearm. She presented to our hospital with her third recurrence of skin lesions in the same location. Histopathological examination of the skin revealed the features of BCC with evidence of perineural invasion (PNI). She underwent a margin-free, wide local excision, vacuum-assisted closure of the wound, and reconstructive surgery using a skin graft. She also underwent a sentinel lymph node biopsy (SLNB), which was negative for the tumor. Then she was referred for radiotherapy. Although the patient underwent a free-margin excision in the previous episodes, she came back years later with a recurrent lesion.

It is considered that basal cell carcinomas larger than 3 cm in size with perineural invasion evidence on histopathological examination and with deep tissue involvement have a bad prognosis compared with the smaller superficial lesions. Thus, based on the findings in our case and the previously reported cases, careful follow-up is recommended for patients with BCC with bad prognostic factors. In certain high-risk cases, SLNB should be considered to rule out occult metastasis.

## Introduction

Basal cell carcinoma (BCC) is the most commonly diagnosed skin malignancy in the world [1]. The global incidence of BCC is increasing annually, and there is evidence that the frequency of aggressively growing BCC has also been increasing, which can cause significant local tissue destruction that necessitates more complex treatment and investigations [2]. Herein, we report a case of recurrent BCC with aggressive histopathological features and discuss the high-risk group for developing aggressive lesions.

## Case presentation

A 36-year-old lady presented with a skin lesion on her right forearm. The lesion had a waxy color and ill-defined borders. She also complained of increasing itching over the area of the lesion. She had a history of BCC in the same location, so she sought medical advice at our clinic as soon as she noticed the skin changes. The lady had no risk factors for BCC, and no sun exposure was reported.

The lady first noticed the lesion seven years ago; it was about 4*4*3.5 cm in size, and located on the volar aspect of the right forearm. The lesion was red in color and itchy. She ignored it for three years, as she thought it was a lesion that might not need any medical attention. However, the lesion gradually increased in size, and the itching became annoying, so she sought medical advice at a hospital. A biopsy was done and referred for histopathological examination, which showed adenoid BCC. The patient was referred for a wide surgical excision of the lesion. The histopathological examination of the excised tissue confirmed the previous results of the biopsy but also showed positive margins. Thus, the patient was referred for re-resection with negative margins. The patient underwent excision; the margins were free of tumor, and the site was covered with a skin graft.

**Figure 1:**
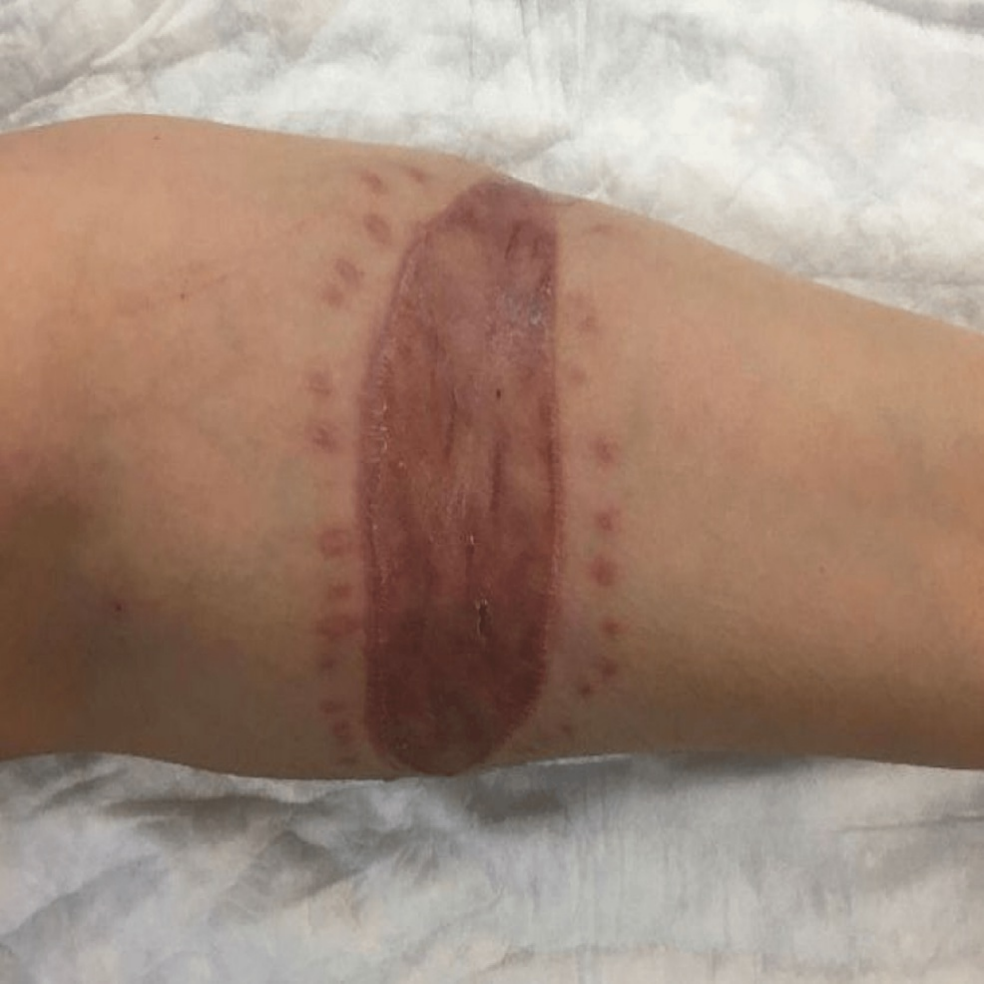
The lesion of the grafted skin on the right forearm at the first presentation to our hospital

**Figure 2:**
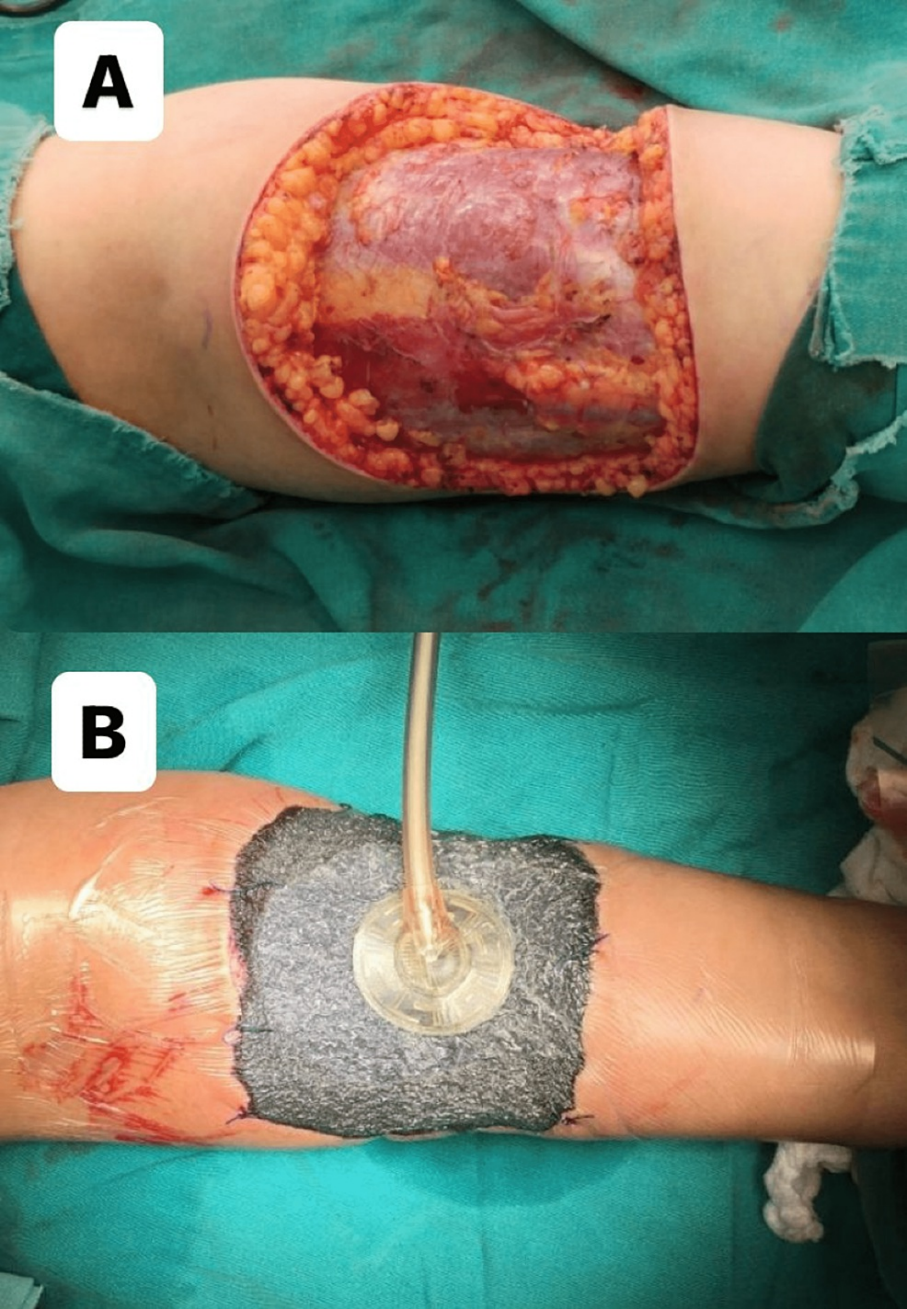
The first surgical resection; (A) wide and deep surgical resection, (B) vacuum device application to the excision area

Six months later, the patient sought medical advice at our hospital, complaining of skin discoloration that looked waxy with severe itching in the grafted area (Figure 1). Multiple biopsies were taken from the skin at the periphery of the skin graft, and they were positive for adenoid BCC. A wider resection of the lesion was performed with 1 cm free margins and depth based on the results of the frozen section with the conservation of the deep fascia. Negative pressure wound therapy was applied and was changed every four to five days until good granulation was noticed after four weeks. Then, she was referred for reconstructive surgery, and a 12*9 cm split-thickness skin graft was used to cover the site of surgery (Figure 2). The final histopathological examination showed negative margins on the excision.

**Figure 3:**
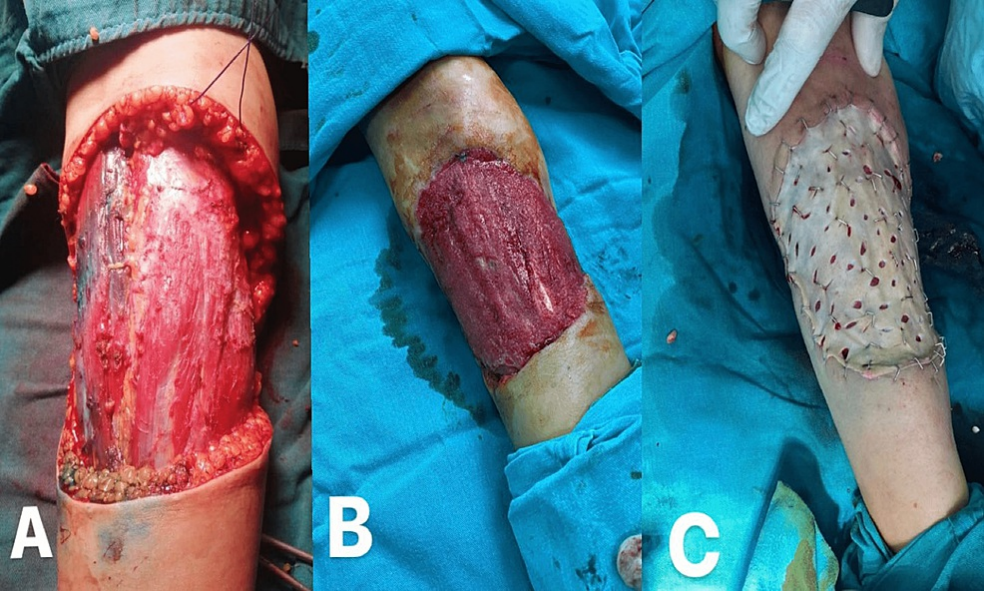
The second surgical excision; (A) the excision area, (B) the area of excision after vacuum application and granulation tissue formation, (C) wound reconstruction by full thickness skin graft

**Figure 4:**
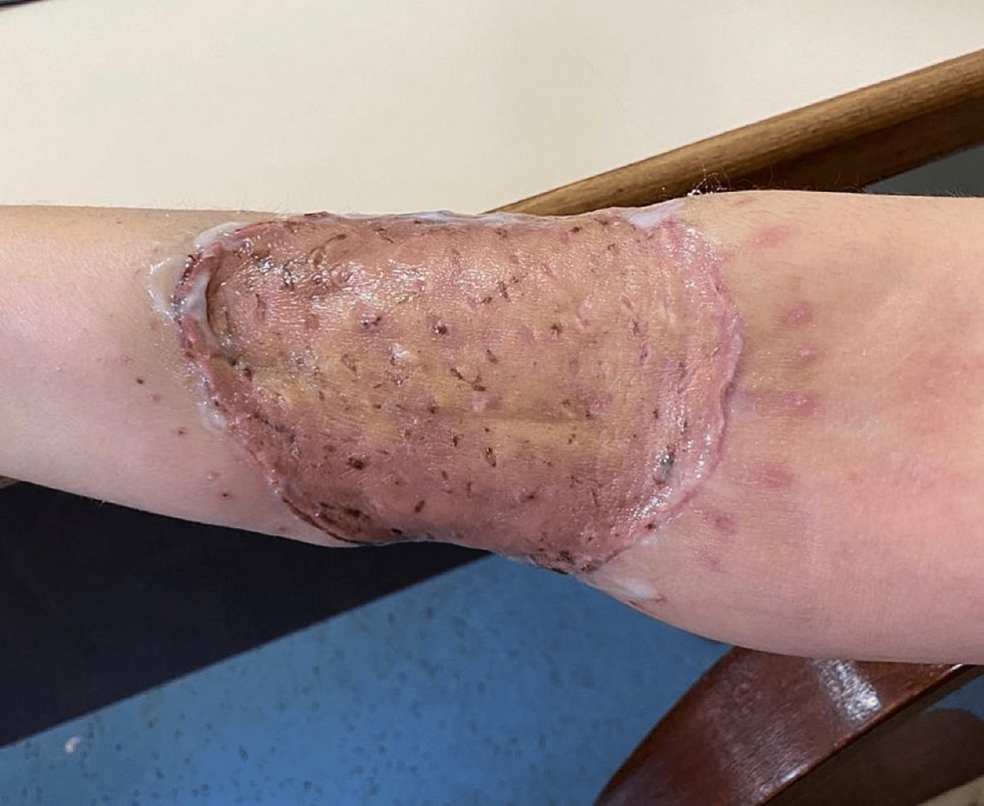
Good healing of the second reconstructive surgery wound

**Figure 5:**
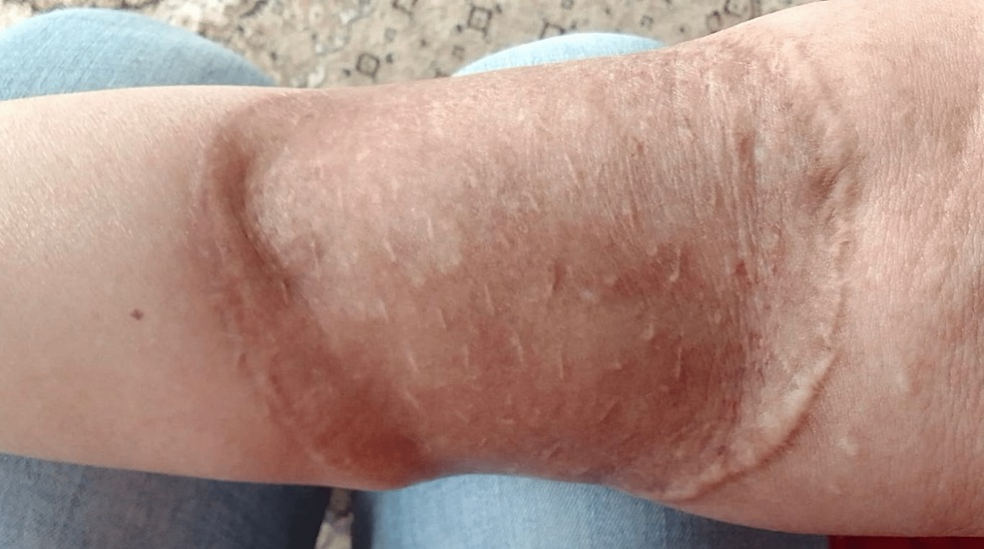
The site of excision and reconstruction two years after surgery and after 30 sessions of radiotherapy (60 gray)

Three years later, the lady sought medical advice at our hospital with skin changes and itching over the site of the grafted skin, similar to what she had experienced before. On examination, the skin looked healthy, and the scars from the graft were well healed. However, a skin biopsy was taken from the suspected site on the right forearm at the margin of the previous excision site, containing normal skin and skin from the graft. The histopathological examination of this biopsy showed recurrent basal cell carcinoma with perineural invasion (PNI). After a discussion of a multidisciplinary team of oncologists, general surgeons, and plastic surgeons, multiple random biopsies were taken from the circumference of the diseased area to rule out other positive areas and assess the need for extending the area of excision. The patient was referred for wide local excision of the area that was proven positive, which included the deep muscle fascia and a 1.5 cm free margin. The operation also included surgical vacuum placement to accelerate the granulation tissue formation. In addition to this, a right axillary sentinel lymph node biopsy (SLNB) was done; the number of lymph nodes was adequate, and it was negative. Fibrofatty tissue was extracted, and it was negative for the tumor as well. Four weeks after the use of the vacuum, reconstructive surgery was performed using a full-thickness skin graft taken from the left inguinal area that was closed by primary closure after undermining (Figure 3). The patient was then referred for radiotherapy after ensuring good healing of the wound (Figure 4). She had 30 sessions of radiotherapy at a dose of two grays per session (Figure 5).

## Discussion

The world-wide incidence of BCC is increasing annually, and more cases have been reported to cause significant tissue destruction [1,2]. The risk of developing BCC has been associated with multiple factors. Ultraviolet (UV) radiation exposure is the primary environmental risk factor for BCC; other risk factors include increasing age, family history, chronic use of immunosuppressants, HIV infection, arsenic exposure, and ionizing radiation [3]. BCC's association with UV radiation exposure contributes to making the sun-exposed areas more susceptible to developing the lesion. The studies report that the most common anatomical location of BCC is the head, with the highest percentages on the nose, but the lesion may occur anywhere on the body [2,4]. In our case, the lesion was noticed in the anterior aspect of the forearm, which is not a common location for the lesion and not a location with continuous exposure to the sun.

The lady in our case was 30 years of age when she was diagnosed with BCC for the first time, and despite the fact that increasing age has been known to be a major risk factor for BCC since ages, recent studies show that the incidence of BCC is increasing over time in the young population, particularly in women [5].

The frequency of aggressive BCC has been increasing over the years, and this necessitates more complex treatment and investigations. Aggressive clinical behavior of BCC can be presented as local tissue destruction, loco-regional recurrence, and metastasis. The histopathology of the lesion is related to the clinical picture and is very helpful in determining the therapeutic plan and the prognosis. Generally, the presence of perineural invasion (PNI) is a major microscopic finding that portends aggressive clinical behavior [6]. In our case, the histopathology report of the biopsy from the lesion developed in the third episode showed evidence of perineural invasion, which was a hint for us to expect the behavior of the lesion and to do more investigations, including the sentinel lymph node biopsy (SLNB) and the radiotherapy referral.

The rate of metastatic basal cell carcinoma (MBCC) is less than 1%, and it usually metastases to the regional lymph nodes. The rate increases to approximately 2% in BCC greater than 3 cm in diameter [7,8]. Some factors other than tumor size have been identified to portend lymphatic metastasis; these include deep soft tissue invasion, perineural invasion (PNI) on histopathology, and specific histopathological subtypes such as the morpheaform and the infiltrative subtypes [2,6,9]. A previous study encouraged examining the nearest lymph nodes with prophylactic lymphadenectomy in histologically aggressive cutaneous malignancies or in the presence of perineural or local soft tissue invasion [10]. In our case, when the lesion was noticed, it was 4*4*3.5 cm in size, which is an indicator of aggressiveness. However, the histopathological subtype was described as adenoid, which is a benign and rare variant of the nodular BCC [11]. The size alone, besides the benign histopathology and the superficial involvement of the surrounding tissue, made the surgical excision and the close follow-up sufficient. In the second attack, which was six months from the last surgery, there was no other indicator for aggressiveness other than the tumor size. However, a deep excision was performed to ensure the absence of any occult tumor cells. The plan was agreed upon by the multidisciplinary team involved in this case.

In the third recurrence, which was three years after the last one, there was a larger, deeper involvement of tissue, and the histopathological examination showed perineural invasion (PNI). The size of the skin lesion, the frequency of recurrence, the depth of the involved tissue, and the histopathological examination made us suspect an aggressive BCC. SLNB has been used for detecting occult metastasis in regional lymph nodes in various cancers, and there have been a few reported BCC cases with lymph nodes and distant metastasis. For example, an infiltrative growth pattern BCC on the forearm of an 83-year-old woman, with an invasion of the surrounding neurovascular structures, was found to cause a positive spread to a lymph node (LN) in the deltopectoral groove [12]. In another study, a 63-year-old man who had a 4 cm basosquamous cell carcinoma (BSC) on the back was referred after a positive SLNB biopsy to undergo a therapeutic complete lymphadenectomy and was found to have five out of 14 positive LN [13]. Another study of a 69-year-old man who had a BSC on his left thigh and was referred for lymph node examination found a regional lymphatic spread of the tumor [14]. With all of these studies and the existence of multiple indicators for aggressiveness in our case, we proceeded with the decision to take SLNB.

Careful follow-up is recommended for patients with lesions classified as high-risk for aggressive clinical behavior. The lady in our case presented to our clinic after three years of the last recurrence of BCC with the same lesion, which is not so long compared to what we expect from BCC as it is considered a slow-growing cancer. However, when BCC shows signs of aggressive behavior, we are not surprised by the speed of recurrence. A randomized clinical trial with 10 years of follow-up showed that 44% of BCC recurrences occur within the first five years post-treatment. Thus, we recommend annual close follow-up for patients with high-risk BCC after recovery [15].

## Conclusions

It is known that BCC occurs most commonly in the head and among the elderly, but it can also occur anywhere else in the human body and at a young age. Thus, when suspecting a BCC lesion in an unusual presentation, a biopsy should not be delayed.

It is considered that basal cell carcinomas larger than 3 cm in size with perineal invasion evidence on histopathological examination and with deep tissue involvement have a bad prognosis compared with the smaller superficial lesions due to the aggressive behavior that is expected from the tumor. Aggressive clinical behavior of BCC can be presented as local tissue destruction, loco-regional recurrence, and metastasis. Thus, based on the findings in our case and the previously reported cases, careful follow-up is recommended for patients with BCC with bad prognostic factors to early detect any recurrence. Additionally, in certain high-risk cases, SLNB should be considered to rule out occult metastases.

Multidisciplinary team (MDT) meetings can be an effective method to deliver comprehensive care for patients by facilitating a group discussion among healthcare professionals from different disciplines to give them a chance to use the best of their knowledge and clinical skills to improve outcomes. We recommend considering MDT meetings regularly at hospitals and health care centers.
